# The Role of Radiomics in the Era of Immune Checkpoint Inhibitors: A New Protagonist in the Jungle of Response Criteria

**DOI:** 10.3390/jcm11061740

**Published:** 2022-03-21

**Authors:** Angelo Castello, Massimo Castellani, Luigia Florimonte, Luca Urso, Luigi Mansi, Egesta Lopci

**Affiliations:** 1 Nuclear Medicine Unit, Fondazione IRCCS Ca’ Granda, Ospedale Maggiore Policlinico, 20122 Milan, Italy; massimo.castellani@policlinico.mi.it (M.C.); luigia.florimonte@policlinico.mi.it (L.F.); 2 Nuclear Medicine Unit, Oncological Medical and Specialist Department, University Hospital of Ferrara, 44121 Ferrara, Italy; luca.urso@unife.it; 3 Interuniversity Research Center for the Sustainable Development (CIRPS), 00152 Rome, Italy; luigi.mansi@libero.it; 4 Nuclear Medicine Unit, IRCCS—Humanitas Research Hospital, 20089 Rozzano, Italy; egesta.lopci@gmail.com

**Keywords:** radiomics, texture analysis, deep learning, immune checkpoint inhibitors, lung cancer, PET/CT, response assessment, survival

## Abstract

**Simple Summary:**

The introduction of immune checkpoint inhibitors has represented a milestone in cancer treatment. Despite PD-L1 expression being the standard biomarker used before the start of therapy, there is still a strict need to identify complementary non-invasive biomarkers in order to better select patients. In this context, radiomics is an emerging approach for examining medical images and clinical data by capturing multiple features hidden from human eye and is potentially able to predict response assessment and survival in the course of immunotherapy. We reviewed the available studies investigating the role of radiomics in cancer patients, focusing on non-small cell lung cancer treated with immune checkpoint inhibitors. Although preliminary research shows encouraging results, different issues need to be solved before radiomics can enter into clinical practice.

**Abstract:**

Immune checkpoint inhibitors (ICI) have demonstrated encouraging results in terms of durable clinical benefit and survival in several malignancies. Nevertheless, the search to identify an “ideal” biomarker for predicting response to ICI is still far from over. Radiomics is a new translational field of study aiming to extract, by dedicated software, several features from a given medical image, ranging from intensity distribution and spatial heterogeneity to higher-order statistical parameters. Based on these premises, our review aims to summarize the current status of radiomics as a potential predictor of clinical response following immunotherapy treatment. A comprehensive search of PubMed results was conducted. All studies published in English up to and including December 2021 were selected, comprising those that explored computed tomography (CT), magnetic resonance imaging (MRI), and positron emission tomography (PET) for radiomic analyses in the setting of ICI. Several studies have demonstrated the potential applicability of radiomic features in the monitoring of the therapeutic response beyond the traditional morphologic and metabolic criteria, as well as in the prediction of survival or non-invasive assessment of the tumor microenvironment. Nevertheless, important limitations emerge from our review in terms of standardization in feature selection, data sharing, and methods, as well as in external validation. Additionally, there is still need for prospective clinical trials to confirm the potential significant role of radiomics during immunotherapy.

## 1. Introduction

In the last decade, cancer treatment has been characterized by a growing development of new therapeutic agents, mostly involving the re-activation of the immune system. In fact, several studies have demonstrated how immune cells interact with malignant cells, inhibiting their growth, and which different mechanisms of immune evasion can be implemented by tumor cells to avoid immune control [1,2]. Immune checkpoints, represented by cytotoxic T lymphocyte antigen 4 (CTLA-4) and programmed cell death protein 1 (PD-1) and its ligand 1 (PD-L1), are the most studied targets of immune escape through the negative regulation of T lymphocytes by tumor cells. Likewise, the discovery and the introduction into clinical practice of immune checkpoint inhibitors (ICI) has revolutionized the therapeutic armamentarium for cancer patients [3]. Ipilimumab was the first ICI approved in 2011 for treating metastatic melanoma. Since then, other ICI have been discovered and analyzed, and currently, nine of those are available on the market for treating approximately 16 different types of cancer. Furthermore, the combination of ICI with other anti-cancer therapies—e.g., immuno-oncology or targeted molecules, chemotherapy or radiotherapy—has been approved, representing more than 76% of all oncology trials [4,5,6].

Before starting ICI therapy, the characterization of the immune profile is required through biomarkers analysis of tumor tissue samples obtained from patients. In particular, genetic mutations, inflammatory cytokines, PD-L1 and CTLA-4 levels, and tumor-infiltrating lymphocytes (TIL) should be assessed, as their expression has been related to a favorable response to treatment. However, a complete prediction of ICI effectiveness is still a challenge, as several factors could influence the therapy outcome, such as intra- and inter-lesion heterogeneity and the progressive modifications induced by previous treatments in the tumor microenvironment (TME). Moreover, an incorrect sampling evaluation can sometimes occur, particularly when evaluating small or highly heterogeneous tissues [7,8,9,10,11].

In the era of personalized medicine, a correct identification of patients who will benefit from ICI is indispensable. Therefore, quantitative image analysis presents a great potential in the pathway of personalizing patients’ management [12]. In this context, radiomics—defined as the process of identifying mineable parameters hidden in the pixel of images and routinely non-detectable with the human eye—could potentially have a rising role. Radiomics is being applied in several fields of medicine, with the aim of defining tumor phenotypes, including grade, TME, gene expression, response to systemic treatment, and prediction of clinical outcomes, as demonstrated by numerous studies involving different malignancies [13,14,15]. Radiomic features present several advantages for clinical oncology application in the near future. First is its non-invasive nature, then the wide availability of medical images, as all oncologic patients undergo several imaging procedures during their disease. Moreover, as medical images show all lesion distributions, the critical limitation of sampling biopsies is overcome. Finally, imaging data have the potential to be used longitudinally in order to track their modifications overtime and potentially to identify treatment-resistant tumors [16].

The aim of this review is to systematically summarize the current radiomic evidence in cancer immunotherapy, particularly focusing on non-small cell lung cancer (NSCLC), by providing in addition a helpful guide for clinicians approaching these new concepts.

## 2. Methods and Materials

Our systematic review was conducted following the Preferred Reporting Items for Systematic Reviews and Meta-analyses (PRISMA) statement [17].

### 2.1. Literature Search Strategy

Potentially relevant publications, eligible for our review, were identified through the PubMed and Web of Science databases. We did not select a start date, but the last update of the literature search was 31 December 2021. We used the MeSH term “cancer” combined with the following keywords: “radiomics”, “immune checkpoint inhibitors”, “magnetic resonance”, “MRI”, “computed tomography”, “CT”, “positron emission tomography”, “PET”. The authors also evaluated all references cited in the retrieved articles.

### 2.2. Selection of Studies

Two authors (A.C., L.U.) worked independently throughout the scientific records screening process, and then selections were combined. After screening by title and abstract alone, full texts were downloaded for the subset of potentially eligible articles. Disagreements were resolved by two other authors (L.M., E.L.). The criteria for excluding studies were as follows: (a) non-English articles; (b) studies focused purely on methodological aspects; (c) studies in animal models; and (d) case reports, poster presentations, letters, and meeting abstracts. 

### 2.3. Data Extraction

Two reviewers (A.C. and L.U.) extracted the information from each included study: publication year, sample size, study population, study design, imaging modality, research question, treatment, software, segmentation, clinical characteristics, imaging features, validation, endpoints, references.

### 2.4. Quality Assessment

**Figure 1:**
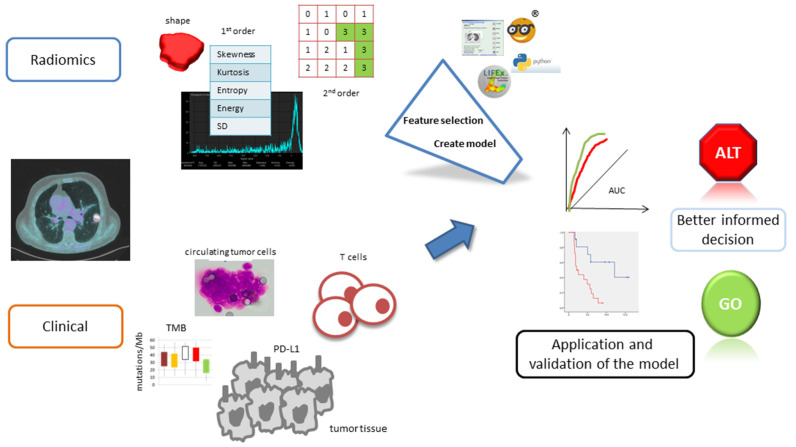
Illustration of the main steps involved in radiomic analysis and model development.

Figure 1 shows the workflow of radiomics in cancer treated by immunotherapy. The methodological quality of the included studies was assessed according to the phase classification criteria for image mining studies [18] and the Radiomics Quality Score (RQS), which is a radiomics-specific quality assessment tool [16].

## 3. Results

**Figure 2:**
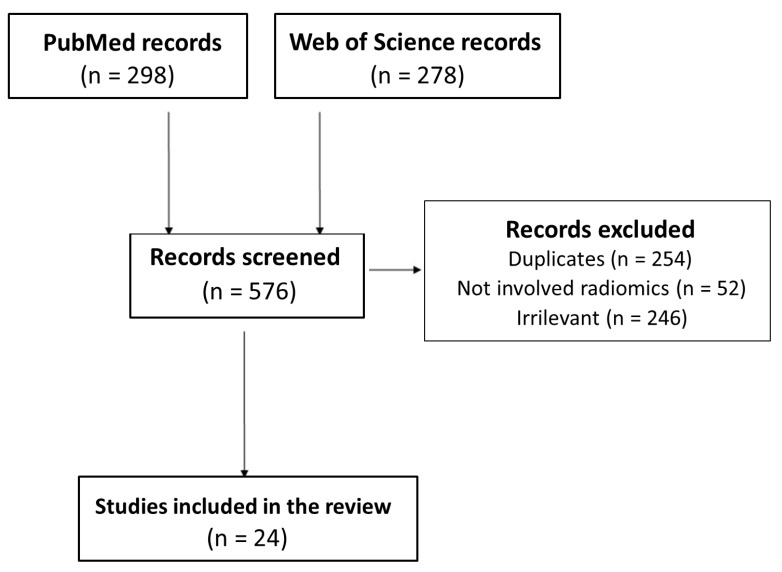
PRISMA flowchart of the study.

Figure 2 shows the PRISMA flowchart of the included studies of our systematic review. The search strategy yielded 298 studies from PubMed and 278 from Web of Science. After exclusion of 552 studies (duplicates, irrelevant titles/abstracts), 24 peer-reviewed articles published were included in this systematic review.

All studies focused on NSCLC except for four studies that also included other tumors. All patients received either anti-PD-1/PD-L1 or anti-CTLA-4 therapy with at least one ICI agent. The most common imaging modality was contrast-enhanced CT (18/24), followed by [18F]FDG PET/CT (6/24) and only one with MRI. There were 20 radiomic studies predicting immunotherapy response or survival, while 4 focused on characterization of tumor immune phenotype.

### 3.1. Quality Analysis 

The number of patients included in the studies in our review ranged from 30 to 399; fifteen studies (62.5%) enrolled more than 100 patients. Most of the studies (22/24, 91.6%) were retrospective, only one study was prospective, and one study was retrospective on training set and prospective on validation set. Only 3 studies (12.5%) performed external validation, whereas 18 studies (75%) performed internal validation, and in 3 studies no type of validation was identified. With respect to phase classification criteria, twelve studies were classified as phase II, seven as phase 0, three as discovery science, and the remaining as phase I (*n* = 1) and phase III (*n* = 1). On the other hand, according to RQS criteria, the mean score of examined studies was 12.2% (range between 0 and 68%), with only three papers characterized by a score greater than 20%.

### 3.2. Non-Small Cell Lung Cancer

As previously mentioned, quantitative image analysis is becoming crucial in the evaluation of clinical outcomes, particularly in oncologic patients. Therefore, the identification of reliable and validated methods for image analysis is of primary importance. While several immune phenotypes have been identified and associated with standard image parameters (i.e., standardized uptake value, SUV; length; volume) [19,20,21], there are still few studies investigating the application of radiomic features as potential predictors of response in patients treated with ICI (Table 1 and Table 2).

One of the first studies in the setting of NSCLC and ICI was from Mu et al. [22], who developed a multiparametric radiomic signature from baseline CT, PET, and PET/CT-fused images for predicting patients with clinical benefit and survival from immunotherapy. In particular, they found that features of heterogeneity, such as short run low gray emphasis or short zone emphasis, were able to predict durable benefit with good results (area under the curve, AUC, was 0.86 for training, 0.83 for retrospective, and 0.81 for prospective test cohorts). Nevertheless, as PD-L1 expression was available only in a few patients, a comparison of their model with the PD-L1 status was not possible, representing an important limitation for the study. Likewise, Ravanelli and colleagues [23] demonstrated on CT images that lung lesions with homogeneous enhancement, expressed by negative values of kurtosis, were less responsive to nivolumab. Intraclass correlation coefficient, ranging between 0.83 and 0.86, demonstrated a good reliability for repeatability of histogram features between the two operators, although retrospective design as well as the absence of an external validation cohort require further studies to confirm these preliminary results. On the other hand, these results were in contrast with those from other studies, which reported worse clinical outcomes in tumors with heterogeneous features by CT texture analysis [24,25]. In particular, Polverari et al. [25] demonstrated that disease progression during ICI treatment in NSCLC was more likely in patients with elevated total lesion glycolysis (TLG), volume, and high tumor heterogeneity represented by asymmetry (e.g., skewness feature) and kurtosis. However, as the study did not establish a simple model for determining clinical application and did not include a robust validation, RQS was low. Similarly, Ladwa and colleagues [26] showed that homogeneous CT texture features, analyzed only in 2D format, were indicative of clinical benefit from ICI treatment when positive skewness was associated with either low entropy (hazard ratio 0.43, *p* = 0.036) or low standard deviation (SD) (hazard ratio 0.42, *p* = 0.04). On the contrary, Shen et al. [27], comparing texture features of contrast-enhanced CT images before treatment, demonstrated that patients with progressive disease had a greater S(2,2)SumEntrp and S(1,0)SumEntrp compared with non-progressive patients, while kurtosis values were lower in the progressive group than in the non-progressive group. Despite the texture features extracted by the classification error probability combined average correlation coefficients (probability of classification error + average correlation coefficient, POE + ACC) model having the best diagnostic efficacy (AUC = 0.812), the 2D image texture analysis may represent an important bias. From these data, they argued that texture features revealed wide CT values and internal structure complexity from malignant lesions, suggesting that a defect of the tumor tissue vascular structure and rich stroma make it difficult for immune cells to penetrate effectively. Recently, a machine learning approach was used to build a model for predicting clinical success of ICI based on CT-radiomic features [28]. Of note, three machine learning classifiers—e.g., support vector machine, logistic regression, and Gaussian naïve Bayes—were demonstrated to be valid for predicting response to ICI, with an AUC value of 0.73 and 0.61 for PFS and OS, respectively, and average Harrell’s concordance indexes for three classifiers of 0.92 and 0.79. Nevertheless, the robustness of the model requires training and testing on a larger data set.

Despite most of published papers having focused on radiomic features at baseline, interesting evidence is also emerging from studies assessing changes in radiomic parameters over time, which seem to be associated with tumor response and survival [29,30,31,32,33]. For example, Khorrami et al. [29] evaluated response to ICI by exploiting differences in CT radiomic features between baseline and after 3–4 cycles of immunotherapy. In a population of 139 patients with NSCLC that was divided into a training set (*n* = 50) and two independent validation sets (*n* = 62, *n* = 27, respectively), their model with eight delta radiomic features discriminated responders from non-responders (AUC of 0.88) and was prognostic for improved OS. Furthermore, combination of perinodular delta radiomics with PD-L1 expression was superior to PD-L1 alone for predicting OS, highlighting the importance of integrating radiomics and biological features into the personalized decision process of NSCLC patients. A similar study, although based on delta features between pre- and post-contrast CT, was conducted by Nardone et al. [30] in 59 patients with metastatic NSCLC treated with nivolumab. According to 14 texture parameters, highly reproducible by ICC analysis (>0.70), the authors differentiated two subgroups at low and high risk for OS, suggesting radiological settings as indirect sign of active immune response. Nevertheless, the arbitrary choice of cut-off values for texture parameters might have biased the study. Similarly, four delta radiomics signatures, extracted by CT images, showed an AUC of 0.77 in the validation set (*n* = 20) for predicting treatment sensitivity to nivolumab. Moreover, PFS was significantly shorter in patients with a high-risk nivolumab signature [31]. Nonetheless, Liu at al [32] developed a nomogram model based on delta radiomics signature, clinical variables, and PD-L1 status that was able to identify responders from non-responders with good accuracy (AUC of 0.83 in the training test). 

Since the introduction of ICI in cancer treatment, several criteria have been proposed for assessing both morphologic and metabolic response. However, there is still uncertainty regarding which method is most suitable for routine clinical use. In this context Valentinuzzi et al. [34] aimed to explore whether [18F]FDG PET/CT radiomic signature (iRADIOMICS) could predict response to pembrolizumab in NSCLC patients. From six primary tumor radiomic features, only higher Small Run Emphasis (SRE) and lower entropy GLCM were able to differentiate responders from non-responders. In addition, SRE and difference entropy showed the highest predictive power (AUC = 0.90) compared with iRECIST (AUC = 0.79 at 1 month and 0.86 at 4 months) and PD-L1 score (AUC = 0.60). In addition, the advent of immunotherapy has revealed new response patterns, such as hyperprogression, dissociated response, and pseudoprogression [35,36,37]. In this setting, Tunali et al. [37] developed a complex predictive model for hyperprogression combining radiomic (approximately 600 features extracted from the largest lung lesions and tumor border regions) and clinical parameters (demographics, mutations, hematologic data) in patients affected by NSCLC treated with single or double ICI. The final radiomic-clinical model obtained, which was based on four clinical covariates and four radiomic features, determined an AUC of 0.80. Specifically, three radiomic features—i.e., radial gradient border SD-2D, border quartile coefficient of dispersion, and border 3D Laws E5E5L5—were extracted from the border regions of the tumors, which is the immediate outside of the tumors and may reflect data related to TME. Despite promising results, the study lacked replication in the independent validation cohorts, which compromised a wider application in clinical routine. Likewise, Vaidya et al. [38] also focused on hyperprogression by radiomic analysis using a model integrating intratumoral and peritumoral texture and vessel tortuosity parameters on baseline CT scans. The random forest classifier distinguished between hyperprogression and other response patterns with an AUC of 0.85 and 0.96 in the training set and validation set, respectively. Moreover, the predicted hyperprogressive patients by the model have a lower OS compared with either responders or non-responders. The quantification of blood vessel morphology, showing that patients were characterized by more tortuous vessel structure in the peritumoral area, was one of the peculiar strengths of the study.

As we have observed from the abovementioned studies, TME is thought to play an important role in aggressive cancers and drug resistance, as well as in chemo- and immunotherapy efficacy. As a matter of fact, different studies have explored the relationship between radiological and/or metabolic uptake features and signaling pathways [30,39]. Similarly, two key studies have investigated the association between radiomic features and T cells infiltration [40,41]. Notably, Tang et al. [40] developed a model, based on the combination of radiomic parameters from pre-treatment CT and tumor immune parameters (i.e., PD-L1 expression and density of TILs) from excised primary NSCLC, for predicting TME. This model divided patients into four clusters and identified a group with longer OS characterized by low CT intensity and high heterogeneity (i.e., low PD-L1 expression and high CD3 infiltration), suggestive of an immune-activated microenvironment. The association of tumor radiomics with immunologic profiles held on multivariate analysis of the training set. The c-index for the multivariate model was 0.70. Likewise, Yoon et al. [41] predicted type 2 helper T cells infiltration with CT radiomic features. In particular, the model that included clinical variables and CT radiomic features showed higher performance compared with that with clinical variables alone (c-indices = 0.646 vs. 0.550). Nevertheless, selection of only patients with PD-L1 information as well as the absence of external validation represent two potential biases of the study. On the contrary, Sun et al. [42] reported a correlation between T cell infiltration and tumors with homogeneous core and heterogeneous peripheries. Their radiomics model was useful for discriminating between immuno-inflamed and immune-desert tumors (AUC 0.76) and for predicting clinical outcomes (i.e., objective response at 3 and 6 months, and OS). Jiang et al. [43], on the other hand, investigated radiomics utility in the prediction of PD-L1 expression, reporting interesting results throughout the application of logistic regression and random forest classifiers, for realizing single predictive models for CT, PET, and PET/CT features. The authors reported that models derived from CT and PET/CT presented the best AUC (0.85–0.97 for 1% level, and 0.77–0.88 for 50% level of PD-L1). However, in the study were employed different machines with different scanning parameters, which may determine potential bias in the analysis.

Tumor-related hypoxia represents another protagonist in the TME, as it is involved with tumor vascularization, growth, invasiveness, metastases, and resistance to therapeutic agents inducing either cell quiescence or an immunosuppressive environment. As a result, tumor hypoxia is associated with clinical outcomes in cancer patients [44]. Recently, Tunali et al. [45] have identified a significant association between gray-level co-occurrence matrix (GLCM) inverse difference and CAIX gene, which is implicated in pH regulation, allowing an acidic TME. Therefore, they suggested a potential application of these biomarkers in the context of immunotherapy, able to identify patients who are unlikely to respond to ICI. Another potential non-invasive biomarker for predicting ICI efficacy has become tumor mutational burden (TMB). He et al. [46] investigated the correlation between deep learning radiomic biomarker and TMB. Using CT images and deep learning technology, they well differentiated high-TMB and low-TMB in NSCLC patients (AUC 0.85 and 0.81 in the training and test group, respectively). In addition, the TMB-radiomic biomarker was able to divide patients as high- and low-risk for both PFS and OS. Hence, although limited to Chinese patients in early NSCLC stage, the study highlighted the role of non-invasive imaging biomarkers for patient selection in the immunotherapy setting.

Although radiomics models have demonstrated a predictive and prognostic value in several cancers, the performance of these models alone is still not enough. In order to improve the prediction of clinical benefits of ICI, the combination of radiomic features with clinicopathological variables has been proposed recently by Yang and colleagues [47]. In a cohort of 92 NSCLC patients, the authors developed two nomogram models, combining radiomic features from baseline CT and clinicopathological variables (i.e., higher Rad-score, younger age, N stage and M stage), identifying with good accuracy (AUC 0.902 in the training cohort) patients with durable response and longer PFS, although without an external validation. Similarly, in one of the largest radiomics studies with 203 patients, Trebeschi et al. [48] used enhanced CT images before treatment to assess the efficacy of ICI in patients with melanoma and NSCLC by an artificial intelligence approach. In particular, their model significantly predicted OS for both tumors (AUC 0.76 and 0.77 for NSCLC and melanoma, respectively. In addition, genomics signature analysis was performed to define the biological substrate of the proposed radiographic biomarkers, revealing a significant association with pathways involved in mitosis. 

Finally, one of the last studies aimed at predicting the risk of cachexia, which is hypothesized to be a cause of ICI resistance. The radiomic signature, based on pre-treatment [18F]FDG PET/CT images, was found to be a significant predictor of cachexia (AUCs ≥ 0.74) and clinical outcomes (AUCs ≥ 0.66) in patients with NSCLC treated with ICI [49].

To summarize, despite the great impulse on radiomics research, we are still far from mature conclusions, and clinical implementations will require further processes. In fact, as demonstrated in a recent meta-analysis, exploring the role of radiomic features to predict response to ICI in NSCLC, most of the studies are characterized by poor methodological quality and scarce reproducibility [50].

### 3.3. Other Tumors

In an analogy to NSCLC, other studies have explored the potential role of radiomic features for predicting response to immunotherapy and clinical outcomes in different malignancies. Nevertheless, almost all studies have been focused on the analysis of TME, investigating CD3 and CD8 tumor-infiltrating lymphocytes in order to evaluate more whether there is a therapeutic window for the application of ICI rather than the actual response to ICI [51,52,53,54,55,56,57]. For example, in a recent study on 45 patients with locally advanced head and neck cancer, the authors investigated whether PET radiomic features could reflect tumor transcriptomics. They identified a significant association between radiomic features and genes involved in cell-cycle, disease, DNA repair, extracellular matrix organization, immune system, metabolism, or signal transduction pathways. Hence, their results suggest a potential role for PET radiomic features in predicting tissue gene expression and, indirectly, personalizing treatment through better patient selection [57]. On the other hand, only Bathia and colleagues [58] evaluated 88 patients with 196 melanoma brain metastases who actually received ICI. According to radiomic analysis of MRI, several features were associated with increased OS. Particularly, higher mean Laplacian of Gaussian resulted in being the most relevant (hazard ratio 0.68, *p* < 0.01), although its clinical significance was lost at multivariate analysis incorporating lactate dehydrogenase and performance status. Similarly, Basler et al. [59] generated seven multivariate prediction models from PET/CT radiomics features, tumor volume, and blood parameters to differentiate pseudoprogression from true progression in 112 metastatic melanoma patients treated with ICI. Of note, higher delta CT coarseness and lower delta CT fractal dimension combined with blood biomarkers (i.e., LDH) showed encouraging results for the early identification of pseudoprogression (AUC 0.82) compared with blood, volume, and radiomics models taken singularly. Even though the study needs external validation, it potentially contributes to a reduction in typical issues during ICI therapy, such as delayed treatment switch or added toxicity.

**Table 1:** Summary of general study features.

Author	Pts	Cancer	Design	Imaging	Timing	ICI	Outcomes	Combination with Non-Radiomics Predictors
Mu [49]	194	NSCLC	Retro-, prospective	PET/CT, CT	Pre-ICI	Anti-PD-(L)1	DCB, PFS, OS	Histology, ECOG, metastases
Ravanelli [23]	104	NSCLC	Retrospective	CT	Pre-ICI	Nivolumab	PFS, OS	NR
Polverari [25]	57	NSCLC	Retrospective	PET/CT	Pre-ICI	Anti-PD-(L)1	RECIST, PFS, OS	NR
Ladwa [26]	47	NSCLC	Retrospective	CT	Pre-ICI	Nivolumab	TTP, PFS, OS	NR
Shen [27]	63	NSCLC	Retrospective	CT	Pre-ICI	Anti-PD-(L)1	iRECIST, PD vs non-PD	NR
Liu [28]	46	NSCLC	Retrospective	CT	Pre-ICI	Nivolumab	PFS, OS	NR
Khorrami [29]	139	NSCLC	Retrospective	CT	Pre-and post 3-4 cycles of ICI	Anti-PD-(L)1	RECIST, OS	Gender, smoker status
Nardone [30]	59	NSCLC	Retrospective	CT	Pre-ICI	Nivolumab	PFS, OS	NR
Dercle [31]	92	NSCLC	Retrospective	CT	Pre-and post 3-4 cycles of ICI	Nivolumab	iRECIST, BOR	NR
Liu [32]	197	NSCLC	Retrospective	CT	Pre-and post 3-4 cycles of ICI	Nivolumab	iRECIST	NR
Valentinuzzi [34]	30	NSCLC	Retrospective	PET/CT	Pre-, 1mo, and 4mo post-ICI	Pembrolizumab	iRADIOMICS	NR
Tunali [37]	228	NSCLC	Prospective	CT	Pre-ICI	Anti-PD-(L)1	hyperprogression	Metastases, prior therapy, NLR
Vaidya [38]	109	NSCLC	Retrospective	CT	Pre-ICI	Anti-PD-(L)1	hyperprogression	NR
Tang [40]	290	NSCLC	Retrospective	CT+tumor immune sample	Pre-ICI	Anti-PD-L1	OS	Lesion size, N-status, histology, age at surgery, prior therapy
Yoon [41]	149	NSCLC	Retrospective	CT	Pre-ICI	Anti-PD-L1	T-cell infiltration	Age, female, smoker status, EGFR+
Sun [42]	135	HNSCC, NSCLC, HCC, BLCA	Retrospective	CT	Pre-ICI	Anti-PD-(L)1	CD8 expression	Tumor volume, prior therapy, Royal Marsden Hospital prognostic score
Jiang [43]	399	NSCLC	Retrospective	PET/CT	Pre-ICI	Anti-PD-(L)1	PD-L1 expression	NR
Tunali [45]	332	NSCLC	Retrospective	CT	Pre-ICI	Anti-PD-(L)1	PFS, OS	Albumin, metastases
He [46]	123	NSCLC	Retrospective	CT	Pre-ICI	Anti-PD-(L)1	TMB	NR
Yang [47]	92	NSCLC	Retrospective	CT	Pre-ICI	Anti-PD-(L)1	DCB, PFS	age, metastases
Trebeschi [48]	123	NSCLC, melanoma	Retrospective	CT	Pre-ICI	Anti-PD-1	RECIST	NR
Mu [49]	210	NSCLC	Retrospective	PET/CT	Pre-ICI	Anti-PD-(L)1	cachexia, PFS, OS	BMI, metastases, ECOG
Bathia [58]	88	Melanoma	Retrospective	MRI	Pre-ICI	Anti-PD-(L)1	PFS, OS	ECOG, LDH
Basler [59]	112	Melanoma	Retrospective	PET/CT	Pre-ICI	Anti-PD-1 ± anti-CTLA4	pseudoprogression	LDH, S100

Abbreviations: BLCA, bladder endothelial carcinoma; BOR, best overall response; DCB, durable clinical benefit; ECOG, Eastern Cooperative Oncology Group performance status; HCC, hepatocellular carcinoma; HNSCC, head and neck squamous cell carcinoma; ICI, immune checkpoint inhibitors; LDH, lactate dehydrogenase; NLR, neutrophils-to-lymphocytes ratio; NSCLC, non-small cell lung cancer; NR, not reported; PFS, progression-free survival; OS, overall survival; TMB, tumor mutational burden; TTP, time-to-progression.

**Table 2:** Summary of radiomic features.

Author	Radiomic Software	Total/Reduced Radiomic Features	Validation	Model Building Test	Phase	RQS (%)
Mu [49]	MATLAB	790/8	Split sample	AIC, HL	III	24 (68.1)
Ravanelli [23]	TexRAD	NR	Cross-validation	Cox proportional hazards	II	10 (27.8)
Polverari [25]	LIFEx	NR	NR	NR	Discovery science	−3 (0.0)
Ladwa [26]	MATLAB	NR	Cross-validation	General model for combining pairs of texture parameters	0	2 (5.6)
Shen [27]	Mazda	NR/10	NR	LDA, NDA, PCA	0	4 (11.1)
Liu [28]	Python	1106/3	Cross-validation	SVM, LR, GNB	0	11 (29.1)
Khorrami [29]	3D Slicer, MATLAB	99/8	Split sample, external	LDR	II	11 (30.6)
Nardone [30]	LifeX, X-Tile	NR	Split sample, external	Texture score	I	3 (8.3)
Dercle [31]	MATLAB	1160/4	Split sample	RF	0	13 (36.1)
Liu [32]	in-house software	402/7	Split sample	LR	II	17 (45.8)
Valentinuzzi [34]	3D Slicer	490/12	Cross-validation	LR	0	13 (36.1)
Tunali [37]	MATLAB	600/409	NR	LR	Discovery science	5 (15.3)
Vaidya [38]	3D Slicer, MATLAB	198/3	Split sample	RF, LDA, DLDA, QDA, SVM	II	11 (29.2)
Tang [40]	3D Slicer, IBEX	12/4	Split sample	Cox proportional hazards	II	14 (38.9)
Yoon [41]	AVIEW	63/8	Internal, bootstrapping	LR	II	15 (41.7)
Sun [42]	LIFEx	84/5	External	LEN	II	18 (50)
Jiang [43]	Python	1744/24	Cross-validation	LR, RF	II	8 (22.1)
Tunali [45]	MATLAB, C++	213/2	External	Cox proportional hazards	Discovery science	22 (61.1)
He [46]	3D Slicer, Python	1688/1020	Split sample	deep learning	II	16 (44.4)
Yang [47]	Python	110/88	Cross-validation	RF	0	14 (37.5)
Trebeschi [48]	NR	5865/68	Split sample	RF	II	11 (31.9)
Mu [49]	ITK-SNAP, MATLAB	1053/9	Cross-validation	LR	II	17 (45)
Bathia [58]	ITK-SNAP, CERR	21/12	Cross-validation	LR	0	7 (19.4)
Basler [59]	Python	344/NR	Cross-validation	LR	II	14 (38.8)

Abbreviations: AIC, Akaike information criteria; DLDA, diagonal linear discriminant analysis; GNB, Gaussian naïve Bayes; HL, Hosmer–Lemeshow; LDA, linear discriminant analysis; LEN, linear elastic-net; LR, logistic regression; NDA, non-linear discriminant analysis; NR, not reported; PCA, principal component analysis; QDA, quadratic discriminant analysis; RF, random forest; SVM, support vector machine.

Nevertheless, we must acknowledge some limitations in this systematic review. Basically, only published articles in English were included, and we did not perform a quantitative analysis of the results due to high heterogeneity of the included studies in terms of methodology for image reconstruction, feature extraction, and algorithms used.

## 4. Discussion

Despite radiomics being a promising tool for response assessment and prediction of survival in patients treated with ICI, by mining more data beyond those traditionally acquired, it has not yet been employed in daily clinical practice [60]. Indeed, the number of ongoing radiomic clinical trials are 142 compared with more than 2000 on ICI. Among these 142, only 18 studies aimed at evaluating the impact of radiomics in immunotherapy (Table 3). To explain why radiomics is still facing difficulties for translation in the clinical arena, scientists should be aware of the potential sources of error in the radiomic pipeline. These errors could concern both strictly clinical methods and more technical issues related to image mining tools. In fact, the majority of studies are based on small cohorts of patients, mostly derived from only one institution. Moreover, they are observational and have a retrospective design, so that standardization of image acquisition protocols is compromised. A further notable methodological deficiency in the current studies is the lack of adequate external validation, which is the essence of the stability of a radiomic model. As a consequence, radiomic features show different cut-off values among studies, and the relative radiomic models cannot be widely generalizable. This might depend also on the absence of radiomic software standardization, which would guarantee the same feature values extracted from the same image when using different software. Another reason for such variability is related to different PET/CT technology and quality of images among nuclear medicine departments, as it was already evident with the SUV parameter. Therefore, before harmonization of data becomes a constant, results should be examined carefully because the robustness of radiomic models on independent data is still unknown [61,62].

In the recent years, some criteria for classifying radiomic studies have been proposed. For example, the Radiomic Quality Score (RQS) is based on 16 items ranging from acquisition parameters to data sharing in order to improve the quality of radiomic research [16]. More recently, some authors [18] have applied the classification used for drug development, i.e., from phase I to IV, to the radiomic models, highlighting that the scarcity of phase III and IV studies precludes their clinical implementation. Moreover, these scores are not free from drawbacks. For instance, although one of the most cited studies in the radiomic field has a high RQS of about 55%, it was still highly biased, as demonstrated by Welch and colleagues [39,63]. Indeed, they showed that tumor volume was highly correlated with three out of four model features proposed by Aerts, suggesting a lack of feature independence. This is a typical example of the so-called “Clever Hans phenomenon” or, in more scientific terms, spurious correlation, which we should keep in mind when we approach to this new world of radiomics and cutting-edge deep learning systems. Table 4 shows the main shortcomings and possible solutions for improving the quality of radiomic studies.

## 5. Conclusions

Radiomics is still a relatively new field in the domain of medical images in the era of big data and machine learning. Despite the present radiomics being limited to cancer research, its future is certainly bright in order to personalize cancer medicine, including tumor assessment in the course of immunotherapy with checkpoint inhibitors. The positive preliminary results of quantitative imaging features in this context, however, require further investigation in prospective cohorts, and randomized clinical trials in the pipeline could provide the necessary information for proceeding toward the method’s validation. When main issues related to closer collaboration among scientists (e.g., physicians, physics, imaging experts, informatics, statistics), standardization, and reproducible software applications, as well as data-sharing are solved, radiomics will grow exponentially and will play the role of protagonist in everyday practice. 

## Figures and Tables

**Table 3:** Summary of the ongoing clinical trials with ICI and radiomic analysis (source: https://clinicaltrials.gov/, accessed on 14 February 2022).

Cancer Type	Trial Identifier Number	Phase/Status	ICI	Radiomics Aim
Lung Cancer	NCT04984148	recruiting	not specified	PD-L1 expression, PFS, OS, pneumonitis
	NCT03305380	completed	not specified	pneumonitis
	NCT04364776	III, recruiting	durvalumab	PFS, OS
	NCT04994795	not yet recruiting	pembrolizumab ± chemo	PFS, OS, DoR, TTP
	NCT04007068	unknown	pembrolizumab	iRADIOMICS vs. irRC
	NCT03311672	withdrawn	pembrolizumab ± RT	AraG PET-CT radiomic analyses
	NCT04541251	II, recruiting	camrelizumab ± chemo	therapy efficacy and decision-making assistance
	NCT04452058	recruiting	not specified	assist surgery, PFS, OS, ORR, CBR
Lung, melanoma	NCT04193956	recruiting	not specified	treatment response, toxicity
Merkel	NCT03304639	not recruiting	pembrolizumab ± RT	pneumonitis
Esophageal	NCT04821765	II, recruiting	tislelizumab ± chemo, RT	pathologic response, OS
	NCT04821778	III, recruiting	not specified ± chemo ± RT	treatment adverse events, pathologic response, OS
	NCT04821843	III, recruiting	not specified ± chemo ± RT (neoadjuvant)	pathologic response, OS
Urothelial	NCT03237780	II, recruiting	atezolizumab ± chemo	changes in tumor
	NCT03387761	I, completed	Ipilimumab ± nivolumab	responders vs. non-responders
Solid tumors	NCT04079283	completed	not specified ± chemo	treatment response
	NCT04892849	recruiting	not specified	tumor tissue pattern
	NCT04954599	I-II, not yet recruiting	multiple	hypoxia

Abbreviations: PD-L1, programmed death ligand-1; PFS, progression-free survival; OS, overall survival; DoR, duration of response; TTP, time-to-progression; ORR, overall response rate; CBR, clinical benefit rate.

**Table 4:** Summary of main issues and possible solutions for radiomic studies.

Limitations	Suggestions
Small cohort from single center	Multicenter clinical trials
Heterogeneous data (“center effect”)	- prospective studies: imaging protocols can be harmonized before data acquisition (e.g., EARL recommendations)
	- retrospective studies: phantom acquisition, post-filtering steps, or ComBat method
Repeatability and Reproducibility	Open-source software packages with detailed description of the workflow used in the studies;
	Compliant with the IBSI guidelines
Results	Both positive and negative should be reported to avoid the misuse of algorithms or excessive generalization of results
Interpretability (“black box”)	Graph-based or visualization tools for improving the interpretability of radiomic results
Model Validation	Preferably performed on external and independent groups, prospectively collected, ideally within clinical trials
Accessibility	Shared databases among different institutions (anonymized), able to be used as validation sets;
	Incorporated into or interfaced with existing RIS/PACS systems

Abbreviations: EARL, EANM Research GmbH; IBSI, image biomarker standardization initiative; RIS, Radiology Information System; PACS, Picture Archiving and Communication System.

## Data Availability

The data presented in this study are available on motivated request to the corresponding author.
